# AI‐Based Intraoral Videography for Automated Dental Inspection and Charting in Children With Mixed Dentition

**DOI:** 10.1155/ijta/5248891

**Published:** 2026-05-28

**Authors:** Manar Abu Talib, Mohammad-Adel Moufti, Raghad Alrachid, Omnia Abu Waraga, Lolwah Aldhafairi, Sara Alfahad, Abdula Alhaddabi

**Affiliations:** ^1^ College of Computing and Informatics, University of Sharjah, Sharjah, UAE, sharjah.ac.ae; ^2^ College of Dental Medicine, University of Sharjah, Sharjah, UAE, sharjah.ac.ae; ^3^ OpenUAE Research and Development Group, Center of Data Analytics and Cybersecurity, Research Institute of Sciences and Engineering, University of Sharjah, Sharjah, UAE, sharjah.ac.ae

**Keywords:** deep learning, intraoral videos, tooth numbering, YOLO

## Abstract

Examination and documentation (charting) of teeth are indispensable yet labor‐intensive processes, especially in pediatric patients, where mixed dentition, anatomical variability, and limited cooperation during imaging present unique challenges. Despite their distinguished potential, existing AI models lack applicability to pediatric dentistry because they are mostly designed for adult dentition and use radiographic images. To fill this gap, we developed an AI‐powered model for automated pediatric dental charting using real‐time intraoral videos, utilizing a YOLO‐based object detection framework. The model was trained to classify 44 tooth types, including primary and permanent dentitions. The dataset is composed of 112,538 frames extracted from 89 intraoral footages of children aged 6–12 years at diverse dental development stages. Of this dataset, 80% was allocated for training and 20% for testing. The model achieved a mAP@0.5 of 0.405, with a precision of 0.495 and a recall of 0.405 across all tooth classes. Notably, the model performed substantially better in detecting primary teeth, achieving a mAP@0.5 of 0.616, compared to 0.255 for permanent teeth, due to the latter’s ongoing eruption and inconsistent appearance. Despite these limitations, this study is a major advancement toward automating pediatric dental charting and will pave the way for future developments in AI applications for pediatric dentistry, facilitating early caries detection for children in schools and in large‐scale public health screening programs.

## 1. Introduction

Dental charting is a fundamental dental care process that entails meticulously documenting each tooth’s position, state, and stage of growth. Accurate charting is important for identifying dental issues, treatment planning, and monitoring the progression of oral health [1]. Unfortunately, this is generally a time‐consuming, error‐prone process that relies heavily on the physician’s knowledge. This is especially prominent in pediatric dentistry, where eruption patterns from primary to secondary teeth increase variability [2]. However, the rapid development of artificial intelligence (AI) presents intriguing methods for automating dental charting, which would speed up, standardize, and improve the accuracy of the process [3, 4].

Previous research has explored the incorporation of AI, machine learning (ML), and deep learning (DL) into adult dental charting utilizing different imaging modalities, including panoramic imaging [5–8] and intraoral photos [9]. Studies have shown that AI can accurately automate tooth recognition, detect conditions like cavities [10], classify restorations [11, 12], and evaluate periodontal health [10, 13], which minimize the restraints of the traditional methods, thereby ensuring consistency of healthcare and ultimately improving the patient’s outcome at lower costs.

Nevertheless, these models are essentially trained on adult permanent dentition datasets and use static images, which are typically captured for older age groups. While X‐rays are used for children, their application is limited due to concerns about radiation exposure [14] and the difficulty of obtaining clear, consistent images from younger patients. Charting becomes more difficult in children due to mixed dentition, anatomical variations in primary teeth, and the inability to capture consistent images due to the limited mouth space and movement [1].

Despite the potential of AI, there is limited research on AI models created exclusively for pediatric dental charting. Existing approaches do not take into consideration the dynamic nature of pediatric oral anatomy or the challenges of real‐time imaging, resulting in a substantial gap in AI’s application to children.

To bridge this gap, we propose an AI‐powered system for pediatric dental charting that uses real‐time intraoral video recorded by intraoral cameras. Unlike prior approaches that utilized static images, our model focuses on real‐time identification while adapting to pediatric‐specific oral anatomy and patient activity. We created a specific dataset annotated to improve model accuracy and generalization, ensuring precise recognition of primary teeth and mixed dentition features.

Our research has made major contributions to the field, including the following:

– To the best of our knowledge, this is the first AI model that utilizes real‐time video footage for automatic charting of primary and mixed dentitions.
– Creating a unique dataset that addresses anatomical variances in children’s teeth.
– Laying the foundation for more accurate and personalized diagnostic tools.

The paper is structured as follows: Section 2 details the methodology for creating and annotating the ground truth images, as well as the training process of the classifier. Section 3 presents the results of the study. Section 4 covers the discussion, and Section 5 concludes the paper with a summary of findings and future directions.

## 2. Methods and Materials

### 2.1. Data Collection/Patient Enrolment

This study has obtained ethical approval from the University of Sharjah Research Ethics Committee under the number REC‐23‐04‐21‐01‐S. The cohort study included 100 patients at the Paediatric Dentistry Department, University Dental Hospital Sharjah (UDHS), who met the inclusion and exclusion criteria described in Table 1, ensuring representation of diverse dental development stages.

**Table 1:** Inclusion and exclusion criteria.

Inclusion criteria	Exclusion criteria
Children age (6‐12 years old).	Children under 5 years old or older than 12 years old.
Children who have primary or mixed dentitions.	Children who have permanent teeth only.
Children who are cooperative and medically fit.	Children who are noncooperative or medically compromised.

**Figure 1:**
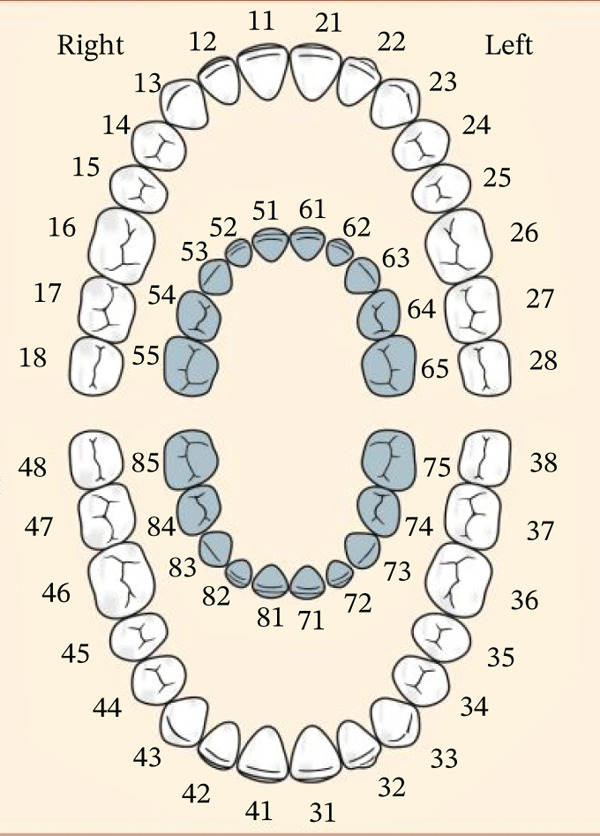
FDI tooth numbering system.

A participation information sheet (PIS) describing the study and its procedures was provided to children and their parents or guardians. To confirm their consent to participate in the study, a consent form was also provided and signed. All pertinent information was gathered, including the gender, year of birth, and medical file number, and kept separately from the video recordings. Data comprised 44 classes corresponding to the mixed dentition listed in Table 2 and shown in Figure 1.

**Table 2:** Classes of tooth IDs and FDI system.

Tooth ID	FDI numbering system
Primary central incisors	51, 61, 71,81
Primary lateral incisors	52, 62, 72, 82
Primary canines	53, 63, 73, 83
Primary first molars	54, 64, 74, 84
Primary second molars	55, 65, 75, 85
Permanent first molars	16, 26, 36, 46
Permanent central incisors	11, 21, 31, 41
Permanent lateral incisors	12, 22, 32, 42
Permanent canines	13, 23, 33, 43
Permanent first premolar	14, 24, 34, 44
Permanent second premolar	15, 25, 35, 45

### 2.2. Data Collection

**Figure 2:**
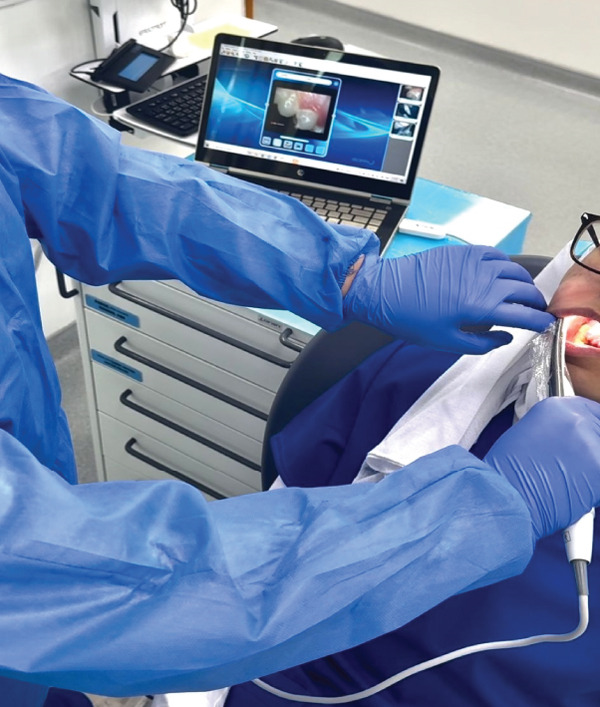
Experimental setup demonstrating the utilization of an intraoral camera for video data collection.

The oral cavities of all participants were filmed using an intraoral camera by three dental students under supervision. Prior to filming, teeth were cleaned to remove any food debris that could compromise the accuracy of the findings. With the camera overseeing the occlusal and lingual surfaces of the teeth, recording began at the most posterior tooth in quadrant one (upper right) and progressed clockwise through each quadrant. Figure 2 depicts the setup and the intraoral camera utilized for capturing video data from patients. The buccal surfaces of the maxillary and mandibular teeth were then recorded with the patient biting. Videos that were unclear upon inspection were marked for retakes, ensuring that only high‐quality records were included in the final dataset. To maintain strict infection control protocols, the intraoral camera was disinfected with alcohol and covered with a protective sleeve before use on each patient, ensuring both patient safety and the integrity of the recordings.

### 2.3. Annotation

Annotation of the captured frames was performed using the Computer Vision Annotation Tool (CVAT, Intel, California, United States), an open‐source software with robust features that enable accurate, collaborative, and efficient dataset construction. The annotation process was performed manually using a bounding box tool to delineate teeth in specific frames and to assign a tooth ID based on the FDI numbering system. To decrease subjectivity and consistent data, teeth were labeled only if they were clearly visible in the frame. Therefore, foggy images or those obscured by such objects as the operator’s fingers or the dental mirror were excluded. Moreover, to maintain consistency across the frames, a tooth’s perspective was reannotated whenever it changed in subsequent frames. For instance, if a tooth was initially labeled from an occlusal view but later appeared from a buccal perspective due to camera movement, it was reannotated to reflect the new angle. In ambiguous or complicated annotation cases, the annotation was discussed among the annotators for a collective opinion. To ensure consistency, a single experienced annotator reviewed the entire dataset. The collected videos were divided into two sets: A training dataset comprising 71 videos (80%) and a testing dataset consisting of 18 videos (20%) were used to develop and refine the model for accurate tooth identification. In total, 112,538 frames, each 640 pixels in size, were captured. Of these, 12,787 were annotated and divided into 3 groups: 8093 for training, 2023 for validation, and 2671 images for testing the performance of the trained model.

### 2.4. Deep Learning

YOLO (You Only Look Once) model has proven its significance in various applications as reliable real‐time object detection and recognition [15]. YOLO models include the following three parts:

1. Backbone, which is responsible for feature extraction and consists of a series of convolutional layers.
2. Neck, which is designed to improve feature representation by fusing multiscale features for better detection. It is extremely useful for objects of diverse sizes.
3. Head, which performs the final predictions, including the bounding boxes, class labeling, and confidence scores.

In this paper, the model YOLOv8 was used as it is equipped with an enhanced feature selection mechanism, such as backbone network enhancement. It assists in a more meaningful feature representation, which improves object identification [16].

**Figure 3:**
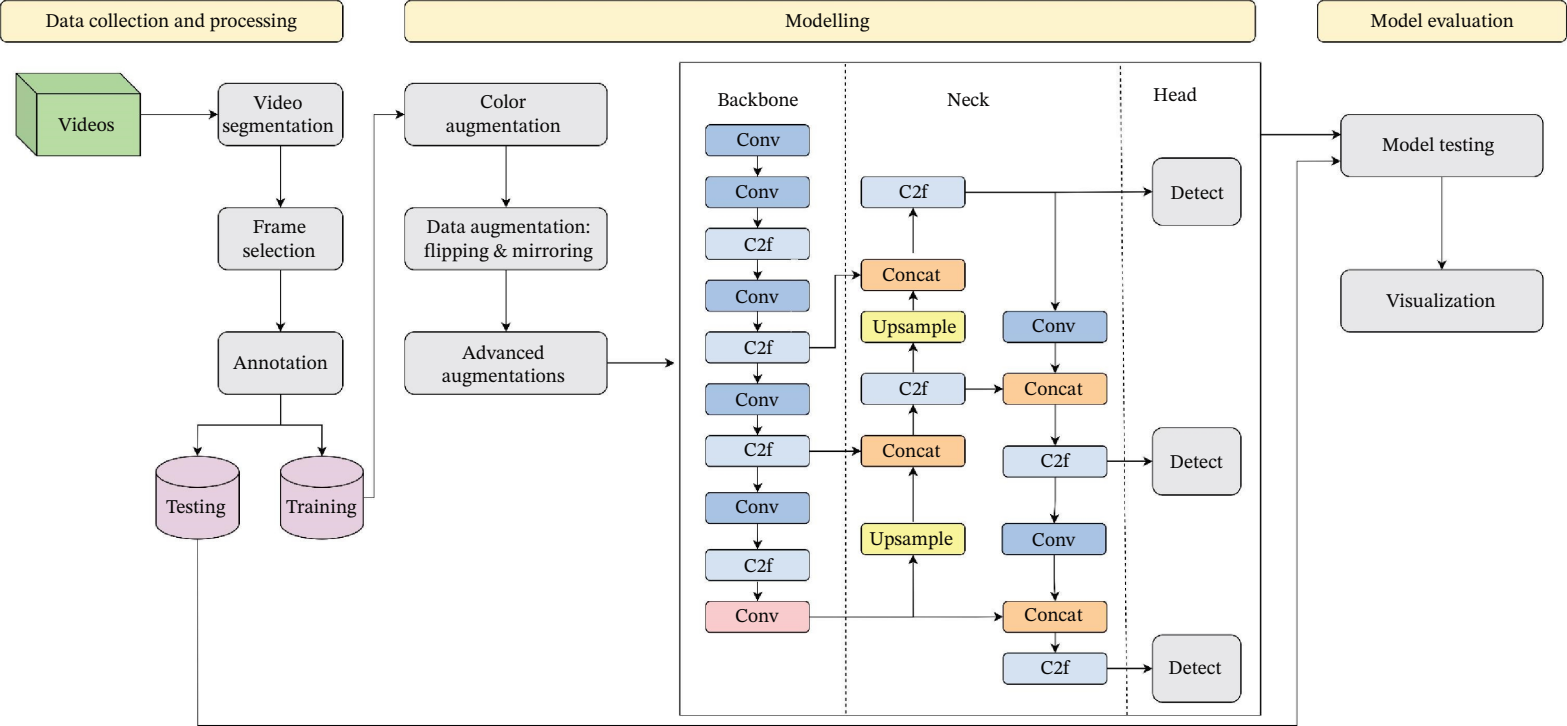
Framework for automated dental video analysis using YOLOv8.

The model was trained using four NVIDIA A100 Tensor Core GPUs, each with a total of 20 GB, for a total of 80 GB, available in our premises to speed up the training; however, this computational setup is not mandatory, and training can successfully be executed on a single GPU with 8 GB. The implementation code used for training and evaluation is publicly available in a GitHub repository [17]. The training dataset was used to transfer learning to the pretrained Yolo8 model and fine‐tune it to match the tooth classes. The hyperparameters used are listed in Table 3. Several augmentation techniques have been implemented. For example, as color augmentation, hue saturation values were adjusted or increased to adapt the model to color variation due to brightness. Moreover, images were flipped vertically and horizontally to improve model robustness and diversity. The model was trained for 50 epochs with 8 frames per batch. A comprehensive overview of the deployed framework is presented in Figure 3.

**Table 3:** Hyperparameters used in the model.

Hyperparameter	Description	Value
hsv_h	Image HSV‐hue augmentation (fraction)	0.0
hsv_s	Image HSV‐saturation augmentation (fraction)	0.5
hsv_v	Image HSV‐value augmentation (fraction)	0.4
degrees	Image rotation (+/− degrees)	0.0
translate	Image translation (+/− fraction)	0.1
scale	Image scale (+/− gain)	0.0
shear	Image shear (+/− degrees)	0.0
perspective	Image perspective (+/− fraction)	0.0 (range: 0–0.001)
flipud	Image flip up–down (probability)	0.5
fliplr	Image flip left–right (probability)	0.5
mosaic	Image mosaic (probability)	0.5
mixup	Image mixup (probability)	0.0
copy_paste	Segment copy–paste (probability)	0.1
erasing	Erases a portion of the image (optional)	Not defined (#)
device	Device indices for computation	[0, 1, 2, 3]

### 2.5. Evaluation Metrics

The model’s performance in identifying each tooth class was evaluated using precision and recall, derived from four key metrics: true positive (TP), true negative (TN), false positive (FP), and false negative (FN). A TP is defined by an acceptable level of overlap between the bounding box drawn by the human investigator and that generated by the model. The overlap, measured using the Intersection over Union (IoU), indicates how well the model’s bounding box aligns with the ground truth box. IoU is calculated by dividing the area of intersection between the two boxes by the area of their union. An IoU ≥ 0.5 is considered “correct.” The higher the IoU, the more accurate the model. If the boxes overlap perfectly, the IoU equals 1.

To further analyze the model’s detection capabilities, a confusion matrix was constructed based on the TP, TN, FP, and FN values. Recall measures the proportion of TPs among all actual positives, while precision reflects the proportion of TPs among all positive predictions.

The average precision (AP) was computed for each tooth class, across recall values ranging from 0 to 1. To provide a thorough assessment of the model’s overall detection ability, the mean average precision (mAP) was then calculated by averaging the AP values over all 44 tooth classes. This mAP score is crucial for assessing the model’s robustness and reliability in real‐world situations. These metrics were computed using the following equations:

Precision=TPTP+FP,

Recall=TPTP+FN,

AP value=∫01PRdR,

mAP value=1n∑i=1nAPi.

## 3. Results

**Figure 4:**
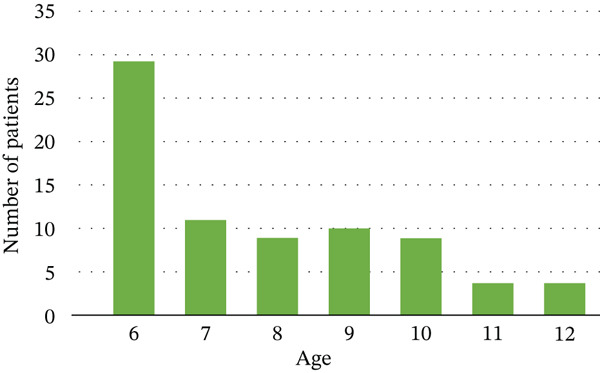
Age distribution.

**Figure 5:**
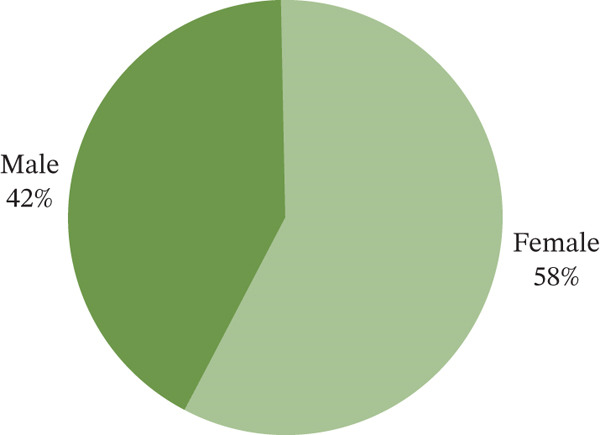
Gender distribution.

The study sample comprised different age groups (Figure 4) and both genders (Figure 5). The largest group is 6 year olds (29 cases).

**Figure 6:**
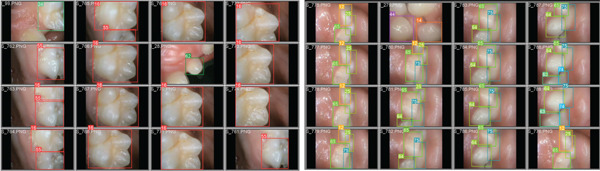
Output of the model on annotated teeth with a bounding box.

The model’s performance was assessed using testing data consisting of 18 videos, which collectively provided 2671 images. Figure 6 presents the visualization results of the trained model. A tooth class is represented by a bounding box, with numerical labels identifying the tooth classes. The figure illustrates the model’s ability to identify and label teeth in frames displaying a single tooth and those with multiple teeth in the same frame.

**Figure 7:**
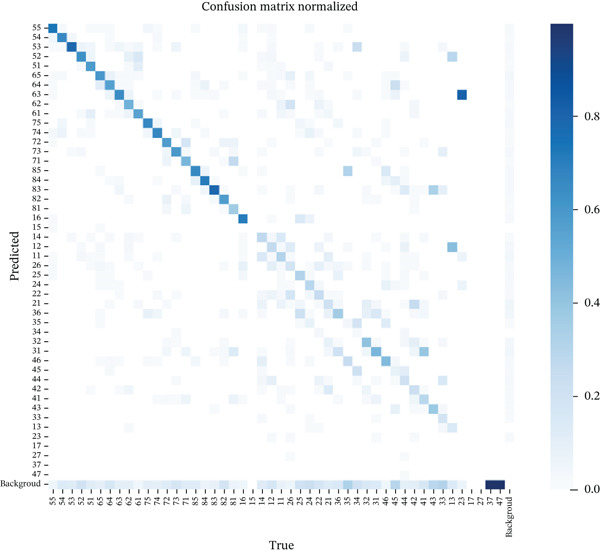
Confusion matrix.

The confusion matrix, shown in Figure 7, provides a detailed comparison between the model’s predictions and the actual ground truth. The diagonal line illustrates the alignment between both classifications. The model accuracy for each tooth class is indicated by the box’s shading, with lighter shading denoting lower accuracy.

**Figure 8:**
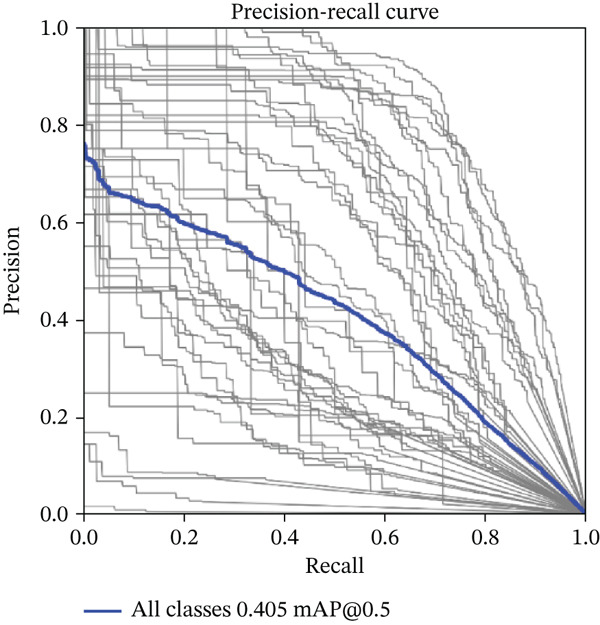
Precision–recall curve.

The accuracy of classification varied considerably among teeth. Notably, primary teeth had a higher accuracy than permanent teeth, as reported in Table 4 and demonstrated by the dark boxes in the top left corner of the confusion matrix (Figure 8). For instance, the primary molar tooth (Class 55) demonstrated the highest accuracy, while the premolar “Class 34” showed reduced accuracy. Instances of misclassification, such as when the model mistaken one tooth type as another or misidentifying soft tissue as a tooth, are shown by the off‐diagonal elements. For example, Class 23, permanent canines, were often misclassified as Class 63, primary canines.

**Table 4:** Model’s performance for each tooth class.

Class	Type	Count of detected objects	Precision	Recall	mAP50	mAP50–95
All	All	6995	0.495	0.405	0.405	0.318
55	Primary U Rt molars	295	0.811	0.725	0.81	0.658
54	Primary U Rt molars	205	0.775	0.637	0.701	0.547
53	Primary U Rt canines	254	0.697	0.791	0.77	0.591
52	Primary U Rt incisors	126	0.626	0.595	0.629	0.493
51	Primary U Rt incisors	84	0.654	0.606	0.621	0.477
65	Primary U Lt molars	277	0.66	0.57	0.643	0.532
64	Primary U Lt molars	198	0.575	0.571	0.533	0.422
63	Primary U Lt canines	289	0.704	0.637	0.655	0.515
62	Primary U Lt incisors	92	0.337	0.465	0.354	0.27
61	Primary U Lt incisors	65	0.339	0.554	0.345	0.256
75	Primary L Lt molars	204	0.755	0.681	0.729	0.566
74	Primary L Lt molars	129	0.553	0.624	0.545	0.394
73	Primary L Lt canines	94	0.612	0.537	0.577	0.422
72	Primary L Lt incisors	252	0.726	0.567	0.682	0.532
71	Primary L Lt incisors	34	0.415	0.529	0.502	0.395
85	Primary L Rt molars	246	0.667	0.646	0.654	0.551
84	Primary L Rt molars	244	0.748	0.695	0.732	0.576
83	Primary L Rt canines	275	0.731	0.767	0.773	0.579
82	Primary L Rt incisors	177	0.74	0.59	0.686	0.523
81	Primary L Rt incisors	30	0.511	0.367	0.398	0.336
16	Permanent U Rt molars	194	0.819	0.701	0.798	0.694
14	Permanent U Rt premolars	137	0.307	0.223	0.183	0.154
13	Permanent U Rt canines	7	0.36	0.429	0.419	0.312
12	Permanent U Rt incisors	232	0.442	0.25	0.271	0.208
11	Permanent U Rt incisors	206	0.379	0.272	0.267	0.213
26	Permanent U Lt molars	197	0.286	0.198	0.193	0.158
25	Permanent U Lt premolars	64	0.244	0.267	0.253	0.214
24	Permanent U Lt premolars	75	0.37	0.267	0.221	0.192
23	Permanent U Lt canines	16	0	0	0.00358	0.00322
22	Permanent U Lt incisors	207	0.356	0.213	0.231	0.181
21	Permanent U Lt incisors	244	0.237	0.205	0.178	0.145
36	Permanent L Lt molars	238	0.34	0.353	0.3	0.234
35	Permanent L Lt premolar	80	0.0782	0.0375	0.0509	0.0405
34	Permanent L Lt premolar	38	0.072	0.0263	0.0232	0.018
33	Permanent L Lt canines	32	0.316	0.156	0.161	0.128
32	Permanent L Lt incisors	214	0.527	0.421	0.428	0.32
31	Permanent L Lt incisors	251	0.395	0.426	0.375	0.272
46	Permanent L Rt molars	160	0.528	0.481	0.473	0.398
45	Permanent L Rt premolars	57	0.129	0.0859	0.0548	0.0439
44	Permanent L Rt premolars	77	0.22	0.221	0.152	0.132
43	Permanent L Rt canines	21	0.286	0.333	0.237	0.165
42	Permanent L Rt incisors	270	0.494	0.23	0.29	0.218
41	Permanent L Rt incisors	168	0.451	0.274	0.308	0.226

∗ U = Upper, L = Lower, Rt=Right, Lt= Left.

Figure 8 presents the precision–recall curve, which illustrates the trade‐off between precision and recall. The curve shows that the model achieved a maximum mAP of 0.405 at a recall value of 0.5. This result highlights the model’s ability to maintain a balance between correctly identifying teeth (recall) and avoiding FPs (precision), but the relatively low mAP score suggests that the model requires further refinement, particularly in addressing misclassifications and improving detection consistency.

Table 4 provides details on the model’s performance for each tooth class. Notably, the model did well in some tooth classes such as Class 55, which had the highest performance, with a precision of 0.811, recall of 0.725, and mAP@0.5 of 0.658. On the other hand, in some classes, the model did not do as well, such as Tooth 23, which had precision and recall, both having zero values and low mAP@0.5 values.

Overall, the model’s performance across the entire dataset was moderate, with precision and recall values of 0.495 and 0.405, respectively. The mAP at an IoU threshold of 0.5, computed by averaging the AP values across all 44 tooth classes, was 0.405.

## 4. Discussion

Tooth numeration is an important dental practice as it plays a key role in standardizing dental status documentation by identifying the type and condition of each tooth. It creates a common language among dentists, making it easier to accurately report patient conditions and communicate them with other dental professionals or related fields. Given that young patients under 12 years old have continuous tooth growth, frequent tracking and documentation of the development of their teeth is critical. However, current manual inspection and charting procedures consume dentists’ time, limiting their availability to spend quality time on treatment and on seeing more patients. Therefore, automated tooth charting tools can help with accurate annotation, optimize diagnostic or screening time, speed up routine tasks, and, in turn, give dentists more time to treat additional patients. Automation can be implemented using radiography or intraoral photography.

Previous studies have been published on tooth detection and identification using DL. A summary of the key studies in this field is presented in Table 5. A significant number of these studies have used panoramic radiographs, which capture the full mouth in a single shot. However, given the risks associated with dental X‐rays [25], radiographs can only be taken in protected and preapproved healthcare facilities. Therefore, despite their high accuracy, radiographs are impractical for schools and public health screening due to their invasive nature and expensive operational costs.

**Table 5:** Summary of related work.

Study	Objective	Model	Dataset	Patient’s age	Tooth type	Results
[18]	Develop and validate an AI‐based system for the automatic detection and numbering of deciduous teeth in pediatric panoramic radiographs to enhance efficiency and accuracy in dental diagnostics	Faster R‐CNN with Inception v2 (COCO) architecture	421 panoramic radiographs	5–7 years	Deciduous teeth (primary teeth)	Sensitivity (true positive rate): 0.9804 Precision (positive predictive value): 0.9571 F1 score: 0.9686
[19]	Develop and evaluate a deep learning model for automated detection and segmentation of deciduous and permanent teeth in mixed dentition PRs, aiming to assist clinicians and reduce error‐prone manual identification	YOLO‐V5	3854 panoramic radiographs	5–13 years	Deciduous and permanent teeth	Detection task: sensitivity, precision, and F1 Score values were all 0.99, with an mAP‐0.5 value of 0.98 Segmentation task: sensitivity, precision, and F1 Score values were all 0.98, with an mAP‐0.5 value of 0.98
[20]	Develop a deep learning model for the automated detection and numbering of both primary and permanent teeth on pediatric panoramic radiographs	YOLO V4	4545 panoramic radiographs	5–13 years	Primary and permanent teeth	mAP at IoU = 0.50: 92.22% for permanent teeth and 94.44% for primary teeth
[21]	Develop an AI model for automatically detecting mesiodens on panoramic radiographs across primary, mixed, and permanent dentition groups, with internal and external validation and a focus on image preprocessing	YOLO v3	612 panoramic radiographs	Primary dentition (3–6 years), mixed dentition (7–13 years), and permanent dentition (over 14 years)	Mesioden (a supernumerary tooth in the anterior maxilla)	Internal test dataset accuracy: 96.2% External test dataset accuracy: 89.8%
[22]	To develop and evaluate object detection methods for identifying dental caries and determining the need for pit and fissure sealing in the first permanent molars, focusing on small object detection and addressing challenges like low resolution and complex backgrounds	YOLOv5 and YOLOX with their respective variants	4563 oral photographs	7–9 years old	First permanent molars	Overall performance YOLOX‐tiling: mAP@0.5 = 72.3*%*, mAP@0.5 : 0.95 = 33.1*%* YOLOv5‐tiling: mAP@0.5 = 70.9*%*, mAP@0.5 : 0.95 = 31.9*%*
[23]	To design and evaluate a deep learning–based AI model for detecting dental plaque on primary teeth and to compare its diagnostic accuracy with that of an experienced pediatric dentist	DeepLabV3+ with transfer learning	886 intraoral photos	5–8 years old	Primary teeth	AI model: Testing dataset: MIoU: 0.726 ± 0.165 Comparison with pediatric dentist dataset: MIoU: 0.736 ± 0.174 For lower resolution photos: MIoU: 0.724 ± 0.159 Pediatric Dentist: MIoU for diagnosing: First diagnosis: 0.695 ± 0.269 Second diagnosis: 0.689 ± 0.253 For lower resolution photos: MIoU: 0.652 ± 0.195
[9]	To develop and evaluate deep learning models for tooth number recognition, detection, and staging of dental caries in intraoral photographic images	Cascade R‐CNN	24,578 intraoral images		Permanent teeth	Tooth number recognition: overall mAP: 0.880 Highest mAP for Tooth 21: 0.975 Lowest mAP for Tooth 48: 0.372 Dental caries detection: Overall mAP: 0.769 Stage 1 mAP: 0.718 Stage 2 mAP: 0.695 Stage 3 mAP: 0.893
[24]	To develop an AI‐based algorithm that classifies images of molar teeth into three categories, that is. full, partial, and nonmetallic restoration automate the dental charting process and aid in disaster victim identification	CNN based on the LeNet architecture	1080 intraoral scans		Molar teeth	Recall: 0.952 (variance 0.000140) Precision: 0.957 (variance 0.0000614) *F*‐measure: 0.952 (variance 0.000145) Overall accuracy: 0.952 (variance 0.000142)

Intraoral photography, on the other hand, has introduced new tools to dentistry such as telemedicine and remote diagnostics, allowing school nurses to conduct regular checkups and detect dental caries at an early stage. This paper is the first to propose the identification of primary and permanent teeth in young patients using intraoral video photography. The proposed solution is highly beneficial for low‐cost oral health screening programs in schools, community centers, and refugee camps, where videos can be collected using intraoral cameras. The developed AI model can be hosted in the cloud to ensure accessibility, while a desktop application running on a laptop or computer can be used to collect video from the camera. The application will securely and anonymously send the videos to the cloud to ensure user privacy. As the developed models are based on YOLO, they operate in real time, which saves time, cost, and resources for healthcare providers. The dental chart generated in the cloud can be exported and shared with parents and healthcare providers. While a few studies applied intraoral still photography, the images were from a single perspective (occlusal) and were limited to one type of dentition, for example, permanent molar or primary teeth only. This limitation helped achieve higher accuracy for the model but limited its generalizability.

This paper is aimed at showcasing the potential of pretrained computer vision architecture in identifying teeth for young patients automatically. The model, autotuned by a self‐collected video dataset, demonstrated the potential of leveraging it in a mobile‐based tooth charting system. The development of such a device represents a positive step toward designing easy solutions that are accessible to users who do not have experience in technology or dentistry. This has been experienced by Xiao et al. [26], who tested the usability of an AI‐based mobile app named AICaries, which detects dental caries for children by their parents at home.

**Figure 9:**
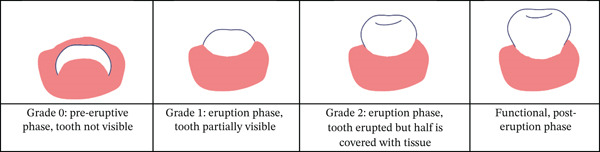
Illustration of the stages of tooth eruption demonstrating the relative shape and position of the premolars.

As evident in the normalized confusion matrix (Figure 7), primary molars and canines were classified accurately, despite their average mAP values, which may be attributed to other factors related to overall classification or precision. Similarly, the primary lateral and central incisors were also classified well despite their lower mAP, which ranged between 0.3 and 0.4 for Teeth 61, 62, and 81 specifically. However, most of the permanent teeth achieved lower accuracy, both in terms of mAP and as reflected in the confusion matrix. This can be attributed to the fact that these teeth are frequently missing or in the eruption stage. As shown in Figure 4, most patients fall within the age range of 6 and 7 years old, which corresponds to the period when primary teeth are commonly present and undergoing transition to permanent dentition [27]. As teeth slowly erupt in the child’s mouth, they exhibit significant changes in size and shape during the eruption stage based on Vandana et al. [28] (Figure 9). This results in significant inconsistencies in the teeth’s “standard” morphology (shape), thus significantly affecting the AI model’s ability to learn and accurately identify teeth in these developmental stages. This may explain why the model did not perform well in identifying permanent teeth’s ongoing eruption compared to the primary teeth that had already completed their eruption in the study group (by the age of 3 years old).

In a prior study, Vandana et al. [28] employed the YOLO model in the tooth classification framework for permanent teeth for intraoral videography, achieving a high mAP score of 78.8%. The model’s performance indicates that the inclusion of both primary and permanent teeth introduces additional complexity in classification, which negatively impacts the detection accuracy.

Another reason the average mAP for primary teeth may be higher than that for permanent teeth is the distinguishable features in size and width between them. The incisors and molars are smaller and shorter to fit within children’s jaws. Additionally, the primary molars are more bulbous than the permanent ones. The reduced mAP50 value for permanent first molars (0.441) compared to their primary counterparts (mAP50 of 0.62), the discrepancy can also be attributed to the fact that permanent first molars are often obscured by the tongue, particularly in pediatric patients with smaller mouths. Consequently, the limited visibility of these teeth poses challenges for accurate detection and classification.

Another notable observation from Table 4 is that permanent canines exhibit the lowest mAP scores among all classes. This can be attributed to the age distribution depicted earlier, specifically, the 11‐ and 12‐year‐old age groups, which typically have a low prevalence of these teeth. As a result, the dataset may lack sufficient samples to adequately train the model to accurately identify and classify them.

Aside from the eruption stage of teeth at an early age, there was noticeable confusion between neighboring and morphologically similar teeth, based on the confusion matrix in Figure 7. Table 6 presents the frequency of misclassification within tooth type, which occurs due to morphological similarity. For instance, Tooth 21 was predicted as 41 and 11, Tooth 14 as 11, Tooth 42 as 21, and Tooth 41 as 31. Some morphologically similar teeth were confused across the left and right sides, such as Tooth 24, a maxillary permanent premolar, which was misclassified as Tooth 35, a mandibular permanent premolar, whereas Tooth 34, a permanent premolar, was misclassified as Tooth 35, a neighboring permanent premolar, or as Tooth 45, a permanent premolar, as they are morphologically similar.

**Table 6:** Frequency of confusion within permanent and primary tooth types.

Tooth type	Central incisors	Lateral incisors	Canines	Molars	Premolar
Primary	32	27	82	105	No applicable
Permanent	171	74	1	40	17

Based on Table 6, many misclassifications have occurred across incisor tooth types. Therefore, Table 7 presents a more in‐depth analysis of the confusion across lateral and central incisors, revealing that misclassifications occurred more frequently with mandibular incisors than with maxillary. Given that both tooth classes have almost the same number of samples in the testing dataset, the higher confusion rate within mandibular incisors may contribute to their morphological similarity. Overall, it is notable that less confusion occurred between mandibular and maxillary teeth, suggesting that the model effectively distinguishes between these two types of teeth. This conclusion aligns with the dental morphology literature, which explains the clear differences between mandibular and maxillary teeth [29].

**Table 7:** Frequency of confusion across permanent incisor tooth types.

Tooth location	Central incisors	Lateral incisors	Lateral with central or vice versa	Total
Incisors mandibular	89	37	72	198
Incisors maxillary	46	28	34	108
Mixing mandibular and maxillary	36	9	No applicable	45

Moreover, the model occasionally confuses permanent with primary teeth, as shown in Table 8. The reverse misclassification occurred less frequently, due to the greater number of primary tooth samples in the dataset compared to permanent ones. For example, some permanent canines were misclassified as primary, such as Teeth 23 and 43, which were misclassified as Teeth 63 and 83, respectively. Based on the table, the majority of the misclassifications occurred between incisor teeth, followed by molars.

**Table 8:** Frequency of confusion between primary and permanent tooth types.

Tooth type	Canines	Incisors	Molars
Primary classified as permanent	7	39	39
Permanent classified as primary	34	158	77

Some classification patterns were observed across tooth classes, but no specific pattern or reason was evident. For example, Tooth 13 was misclassified as Tooth 12, and Tooth 33, a primary canine, was misclassified as Tooth 36, a permanent first molar. Those frequent misclassifications confirm the impact of class imbalance and morphological similarity among teeth, highlighting the limitation of the current backbone in capturing details and identifying the differences. Hence, future work could highlight the benefits of exploring higher input resolution and attention mechanisms in the model’s structure to resolve such confusions.

This highlights the importance of considering age distribution and sample size when evaluating model performance and underscores the need for additional data collection efforts in specific age groups to improve accuracy in tooth detection and classification. The results illustrate the complexity of dental detection in pediatric patients transitioning from primary to permanent teeth. The age group of 10–12 years old was the least represented in the dataset, which directly led to reduced performance. This transitional phase is characterized by variability in tooth morphology and eruption patterns, highlighting the importance of a larger dataset and more balanced data to obtain accuracy and robust results across all ages.

While the moderate performance indicates potential for real‐time pediatric dental charting, it also highlights areas requiring improvement in both the dataset and the model to enhance its ability to handle the variability of pediatric dentition. The development of a more balanced dataset can be further explored by adding more participants or applying data augmentation for minor classes to achieve greater accuracy, particularly for underrepresented tooth classes.

The model’s performance may also be limited by the conditions under which the videos were captured. To enhance its reliability, additional samples or cases should be considered, along with improvements to the setup. This should include additional cases for which we have fewer samples and incorporate samples from individuals of different ages and backgrounds, especially those above 8 years old, to ensure sufficient representation of all classes under all potential conditions.

Moreover, multiple cameras with varying qualities and capabilities, along with different lighting conditions, should be utilized in future evaluations. While the current proposed study did not test the model across different consumer‐grade cameras or real‐world lighting environments, such testing would be very critical to assess the domain shift. Those external factors can positively impact the model’s performance, particularly since data augmentation may not be able to fully capture real‐world environments. This consideration is especially important since the model is intended for use in low‐budget oral health screening programs in schools, community centers, and refugee camps, where clinical lighting and high‐end intraoral cameras might not be available.

**Figure 10:**
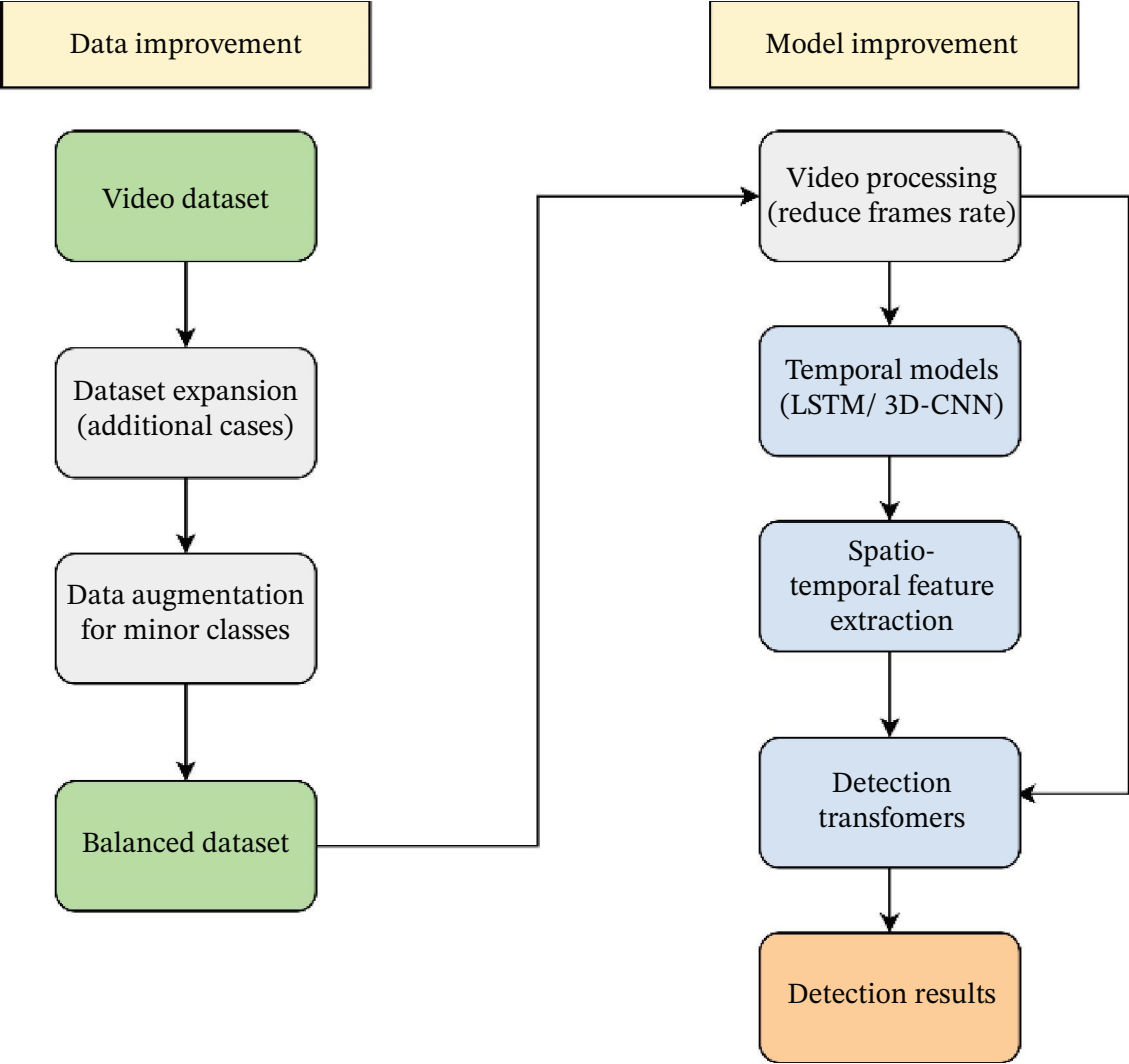
Future roadmap showcasing data improvement and model enhancement.

Moreover, future work should consider integrating temporal modelling techniques, such as LSTM and 3D CNNs, to leverage the video data sequence rather than frame‐wise detection. In addition, detection transformer (DETR), a type of vision transformer, can be explored as it can offer improved understanding of tooth location and appearance. These steps, as illustrated in Figure 10, will enhance the model’s generalization across clinical conditions, reduce the class imbalance effect, and improve model robustness.

## 5. Conclusion

In this study, we developed a YOLO‐based AI model for automated pediatric dental charting using intraoral video data, addressing the gap in AI applications for primary and mixed dentition. Although the model performed well in identifying primary teeth (mAP@0.5 of 0.616), it had difficulties identifying permanent teeth (mAP@0.5 of 0.255) because of their anatomical variability and ongoing eruption in the targeted study sample. The structured annotation process and real‐time video approach ensured a more dynamic and comprehensive dataset. Nevertheless, limitations were noted, such as an unbalanced age distribution, with 29% of the participants being 6 years old. Imaging challenges, such as obstructions from the tongue and patient movements, also affected accuracy. Future studies should focus on generalizing the dataset by encompassing a wider age range, a more diverse population sample, collecting data from different types of intraoral cameras, and examining various lighting conditions. Additionally, efforts should be made to improve imaging techniques to reduce obstacles and enhance video quality. Refining the model to better distinguish between similar tooth types in mixed dentition and adapting it to manage patient movement and cooperation will further enhance its robustness and usability. Despite these challenges, this study is a significant step toward leveraging AI for early caries detection in children and assisting pediatric dentistry.

## Funding

This research was funded by the University of Sharjah, UAE (Grand No. 2301100278).

## Conflicts of Interest

The authors declare no conflicts of interest.

## Data Availability

The data that support the findings of this study are available on request from the corresponding author. The data are not publicly available due to privacy or ethical restrictions.
